# Calcified Right Atrial Thrombus With Near-Systemic Pulmonary Pressures in a Morbidly Obese Patient: A Clinical Conundrum

**DOI:** 10.7759/cureus.34202

**Published:** 2023-01-25

**Authors:** Carlson Sama, Muchi Ditah Chobufo, Li Pang, Joyce Foryoung, James Mills

**Affiliations:** 1 Internal Medicine, West Virginia University School of Medicine, Morgantown, USA; 2 Cardiology, West Virginia University School of Medicine, Morgantown, USA

**Keywords:** outcome, pulmonary embolism, therapeutic anticoagulation, calcified right atrial thrombus, intracardiac mass

## Abstract

Intracardiac masses are not uncommon, but a calcified right atrial thrombus (CRAT) is an exceedingly rare entity and often poses a diagnostic and therapeutic dilemma. We discuss the case of an incidentally noted CcRAT in a 40-year-old man presenting with progressive dyspnea. We further review the literature on the subject, highlighting the need for an individual patient-centred care plan.

## Introduction

Space-occupying lesions in the right atrium are generally rare, with potentially life-threatening differential diagnoses including but not limited to vegetation, myxoma, metastatic disease, cardiac cysts, and a thrombus. The occurrence of a calcified right atrial thrombus is an even rarer manifestation, with very few cases reported globally and little known about its optimal management [1-7]. 

We herein present a unique case of an incidentally noted right atrial calcification and discuss its clinical course, diagnosis, and management in light of current literature. The case should be of interest to physicians in general and, in particular, those practising in the field of thoracic medicine (cardiologists, cardio-thoracic surgeons, and pulmonologists), who are often faced with the clinical conundrum of the best approach to managing such rare entities.

## Case presentation

This is a case about a 40-year-old male with a past medical history of heart failure with preserved ejection fraction (55%), type 2 diabetes mellitus, chronic obstructive pulmonary disease (COPD) on 6L, tobacco use disorder, obesity (BMI 50.14 kg/m2), sleep apnea, obesity hypoventilation syndrome (OHS), deep venous thromboses (DVTs), and pulmonary embolism (PE) on long-term apixaban therapy. He was unsure but estimated having been diagnosed with PEs or DVTs over 10 years ago. He also recounted several DVT episodes during this period, and though he was uncertain, he approximated his last known DVT episode to be about two to three years ago. He presented to the emergency department (ED) of an outside hospital with complaints of worsening shortness of breath (SOB) and bilateral lower limb swelling of one week's duration.

Workup in the ED of the outside facility included initial vital signs of blood pressure 138/83, heart rate 97, respiratory rate 28, temperature 36.7°C, and O2 saturation 96% on 6 L. Arterial blood gas (ABG) showed a pH of 7.43, pCO2 of 38, PO2 of 65, and bicarbonate of 24.9. The chest x-ray showed mild cardiac enlargement and prominent interstitial markings. The ECG showed sinus rhythm with first-degree atrioventricular (AV) block, QTC 488, and cardiac monitoring while in the ED showed short runs of asymptomatic V-tach. Lab work included: troponin at 15.2 and brain natriuretic peptide (BNP) at 695. COVID-19 and influenza A and B were negative. His lactic acid was 1.4, magnesium was 2.0, sodium was 137, potassium was 3.6, chloride was 99, CO2 was 32, blood urea nitrogen (BUN) was 10, creatinine was 1.02, calcium was 9.9, glucose was 120, alkaline phosphatase (ALP) was 113, alanine transaminase (ALT) was 12, aspartate aminotransferase (AST) was 22, total bilirubin was 1.3, white blood cell (WBC) was 7.3, haemoglobin was 13.1, hematocrit was 43.4, and platelets were 151. He admitted to the ED team that he ran out of his routine home medications (including his apixaban, furosemide, and metformin) approximately nine days before the presentation. A tentative diagnosis of acute exacerbation of chronic diastolic heart failure was made, and he was started on IV Furosemide for diuresis, which resulted in a significant symptomatic improvement in his SOB and bilateral lower limb oedema.

**Figure 1:**
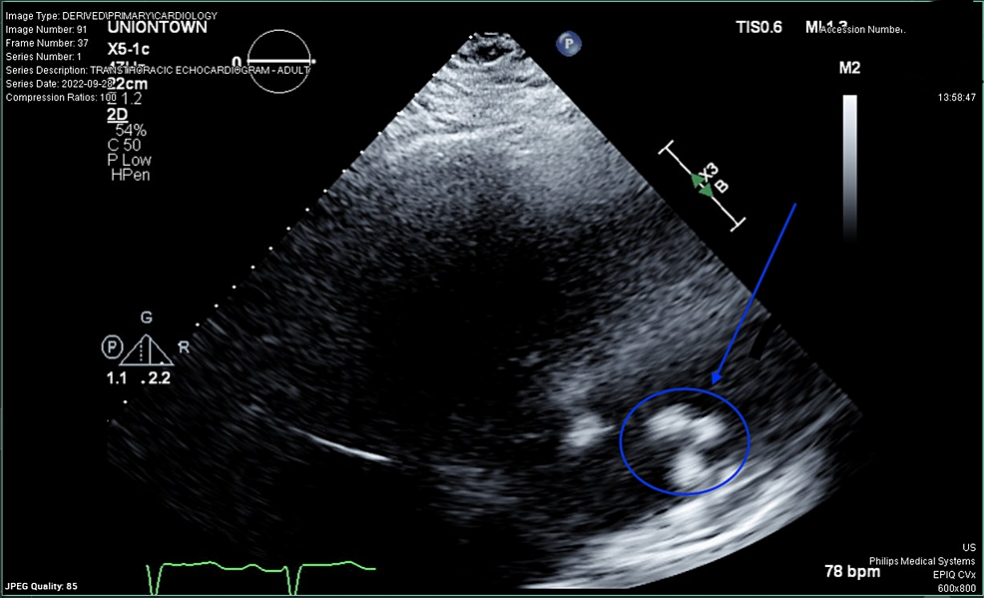
A right atrial mass was incidentally noted on initial transthoracic echography.

**Figure 2:**
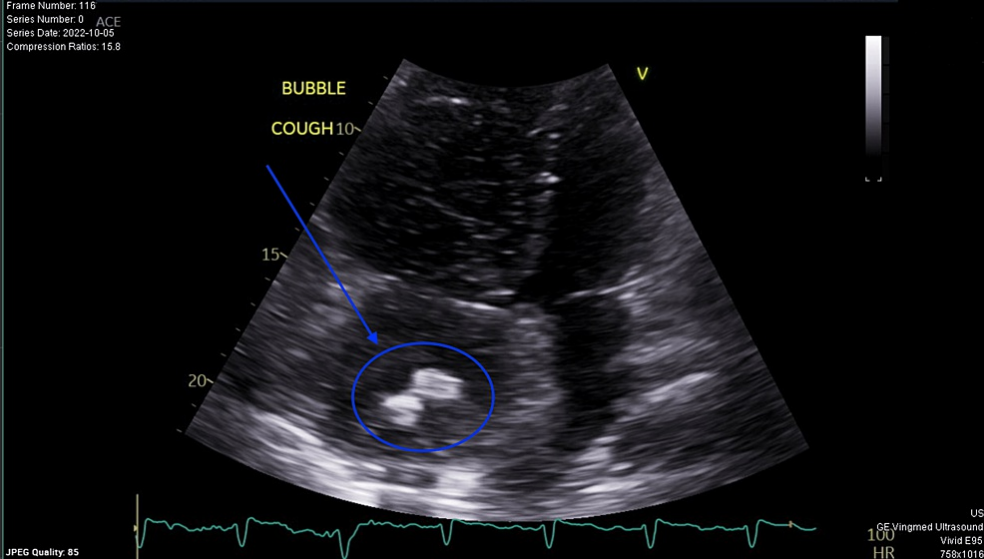
Persistence of right atrial mass post-thrombolysis with alteplase on repeat transthoracic echography.

As part of his initial workup, a transthoracic echocardiogram (TTE) was obtained, which reported a mildly depressed right ventricular systolic function with a right ventricular systolic pressure that is consistent with critical (near systemic) pulmonary hypertension (RVSP 94 mmHg) and a moderately dilated right atrium with an incidental mass found sitting in the right atrium (Figure 1). Due to concerns that the mass may be a thrombus-in-transit, he was started on a heparin drip. An emergent computed tomography angiography (CTA) scan was obtained to evaluate for PE. The CTA scan still showed the known mass in the right atrium, but it was not well demonstrated on the scan. However, a filling defect in the posterior segmental pulmonary artery branches of the left lower lobe, compatible with age-indeterminate thrombus, was noted on the CTA, and no other potential pulmonary emboli were seen. A peripheral duplex scan showed no evidence of DVT within the bilateral lower extremities. Given these findings, the high index of suspicion for an ongoing high-risk right atrial thrombus-in-transit was maintained, and a decision was thus made to initiate thrombolysis with a recombinant tissue plasminogen activator alteplase (rtPA). The patient tolerated the procedure without complications; however, a repeat TTE post-thrombolysis still revealed no change and persistence of the echodensity in the right atrium (Figure 2), raising suspicion for an alternate right atrial mass vs. a thrombus. This prompted the urgent transfer of this patient to our academic tertiary care centre for further evaluation.

**Figure 3:**
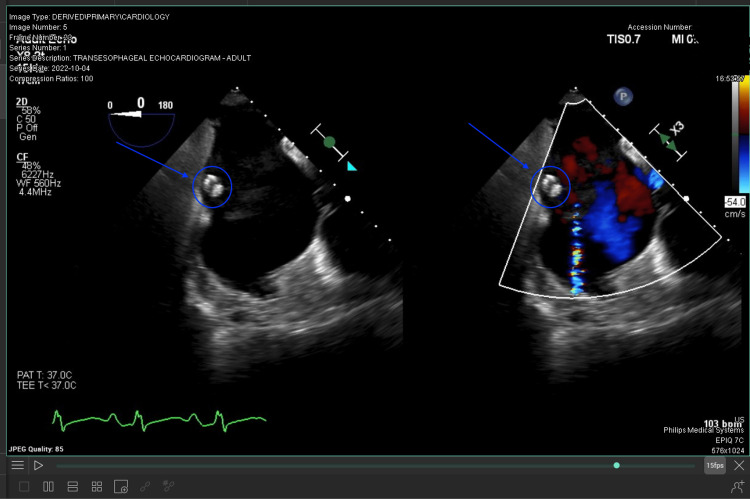
View of a calcified right atrial thrombus via transesophageal echography.

Upon arrival at our facility, he was hemodynamically stable and reported feeling well in himself, with a resolution of his SOB close to the baseline. He denied cough, orthopnoea, paroxysmal nocturnal dyspnea, fever, chills, abdominal pain, nausea, vomiting, or other signs or symptoms suggestive of progressive cardiopulmonary decline. To better define the characteristics of the right atrial echogenicity and interatrial septum, we proceeded to perform transoesophageal echocardiography (TEE), which noted a partially calcified irregular serpiginous/elongated mass with multiple prongs, extending from the superior vena cava into the right atrium and not involving the inferior vena cava, right atrial appendage, or tricuspid valve (Figure 3), most likely avascular on the contrast study, and the entire structure was not visualised on the Definity contrast study. A ventilation-perfusion scan further revealed findings suggestive of bilateral lower lobe segmental or subsegmental pulmonary emboli, and a CT scan of the chest, abdomen, and pelvis was negative for any overt ongoing malignant process. The patient’s clinical status continued to improve with continual diuresis, and he remained hemodynamically stable all along. This increased the likelihood that his initial SOB and bilateral lower leg swelling were an exacerbation of his diastolic heart failure rather than acute right heart failure from an evolving PE.

All images, including the TEE images, were reviewed by cardiac surgeons and interventional cardiologists, and it was determined that the right atrial echogenicity is a thrombus and not a mass. After a series of discussions between both teams, it was concluded that the longevity of the mass could not be determined, but calcification implies that it has been present for an extended period and that the patient would not be an ideal candidate for percutaneous thrombectomy with AngioVac as the mass appears calcified. AngioVac is intended for acute thrombus of fewer than 14 days (soft and pliable). Further, given the mass is stable, attempts at manipulating it with a catheter would be at heightened risk for dislodgement of debris, which may result in significant PE, and we would not recommend attempting it. In addition, given his significant comorbidities, it was believed that the risk of mechanical thrombectomy outweighed the perceived benefit, and he has been deemed a prohibitive risk for cardiac surgery. Both teams agreed the most appropriate treatment at this time should be continuous medical therapy with lifelong anticoagulation with a vitamin K antagonist like Warfarin.

## Discussion

Thrombus formation in the right atrium is a rare event, but when it occurs, it may be associated with deleterious outcomes, including acute right heart failure and pulmonary embolism, with resultant significant morbidity and mortality [8]. Overall, two main types of thrombi have been described in the literature (types A and B). Type A thrombi often originate in the deep venous system, and since they are often free-floating, they carry a high potential to embolize, thus requiring emergency treatment [8,9]. In contrast, type B thrombi are often stationary, fixed to the right atrial wall, and less susceptible to thrombolysis [8,10]. They are uncommon in a structurally normal heart but can however occur in hypercoagulable states, right atrial catheterization, pacemaker implantation, low cardiac output states, septal closure devices, cardiac trauma, cardiomyopathies, indwelling central venous lines, cardiac arrhythmias, neoplastic processes, and some systemic diseases [8,11,12]. They are generally associated with a favourable prognosis given that they are often adherent to the right atrial wall and are less likely to embolize, thus making them a low-risk group compared to type A [3,13]. Table 1 summarises the various aetiologies of intracardiac thrombi, classified according to Virchow’s triad (intracardiac chamber wall, blood flow, and blood components) [14].

**Table 1:** The aetiology of intracardiac thrombi is classified according to Virchow's triad (adapted from Prudhvi et al. [14]).

Cause	Description
Chamber wall causes	Myocardial infarction (akinesis or hypokinesis), dilated left atrium (diastolic dysfunction), dilated right atrium (pulmonary arterial hypertension), ventricular aneurysms, dilated cardiomyopathy, Takotsubo cardiomyopathy, peripartum cardiomyopathy, myocardial non-compaction, endocardial injury due to central venous catheters, pacemakers, defibrillator leads, left ventricular assist devices (LVAD), and atrial septal aneurysms
Abnormal flow states	Heart rate or rhythm disturbances (atrial fibrillation, atrial flutter, ventricular fibrillation, or ventricular tachycardia), increased turbulence due to prosthetic valves, valve stenosis (mitral, tricuspid, or aortic), and mitral annular calcification
Blood component causes	Hypercoagulable states, protein C and/or S deficiency, antiphospholipid antibody syndrome, and paraganglioma due to catecholamine excess

Our patient’s thrombus was neither attached to the atrial wall nor was it freely mobile; it was stationary, calcified, and resistant to thrombolysis with rtPA (alteplase). Given his subjective over 10-year history of recurrent DVTs and an incidental finding of age-indeterminate bilateral segmental and subsegmental pulmonary embolisms, we hypothesised that his right atrial thrombus may have originated from chronic venous emboli that became entrapped in the right heart and subsequently underwent calcification over time. In the setting of a structurally normal heart, no history of invasive procedures, a negative initial workup for a potential malignancy, and a positive family history of blood clots (both his parents), we suspect a primary hypercoagulable disorder as the underlying causative factor for his clots. We also believe that his severe pulmonary hypertension (RVSP 94 mmHg) was mainly group-four-related (chronic thromboembolic pulmonary hypertension (CTEPH)), with some contributions from other group factors, e.g., COPD, obesity hypoventilation syndrome (OHS)/obstructive sleep apnoea (OSA), amongst others.

Though histologic tissue examination is the most reliable way to differentiate chronic calcified intracardiac thrombus from other causes of intracardiac calcification [12], transthoracic echocardiography (TTE) represents a valid first-line imaging study to quickly provide information on the location, echogenicity, and morphology of a thrombus [15]. It is however limited in its ability to clearly differentiate myxomas from mural thrombi, assess an invading cardiac mass, perform tissue characterization, overcome body habitus, and have a restricted field of view [16]. These are difficulties that can be mitigated with the use of transoesophageal echocardiography (TEE) due to its higher sensitivity and specificity [10,16-18], thus accounting for its use in our patient. The role of multimodal advanced imaging (cardiac computerised tomography and cardiac magnetic resonance imaging) is well established in such settings. However, cardiac MRI is superior to cardiac CT scanning in part due to its ability to discriminate between a true cardiac mass and a pseudo-mass. It can further distinguish a cardiac neoplasm from other conditions and provide useful information as to the extent of invasion into the cardiac as well as adnexal structures [16]. All in all, cardiac MRI can be the single best study to obtain complete information, especially if a surgical intervention is being considered.

Various treatment options have been proposed in the management of a right atrial thrombus, including watchful waiting, anticoagulation, thrombolysis, minimally invasive techniques, and open surgery [19]. However, treatment remains controversial as there is no clear consensus on the optimal option, especially in the case of a calcified right atrial thrombus given the rarity of the condition, and no definitive evidence comparing the efficacy of anticoagulation versus surgical resection. Some authors and expert opinions suggest a decision on whether to treat and the mode of treatment should be individualised taking into consideration the extent, size, shape, and mobility of the thrombus as well as pre-existing PE or DVT, cardiopulmonary reserve, and, of course, the patient’s preferences [3, 19].

It is important to recall that our patient had been on long-term apixaban (>five years) but continued to have DVTs during that period. He was thus determined to have failed apixaban therapy. Further, he was treated with thrombolytics (alteplase) at the outside facility before transfer to our facility without any change in the size, shape, position, or form of the thrombus; this suggests an age-indeterminate chronically calcified thrombus that is potentially stable and not a thrombus-in-transit, which appears to reflect findings on TEE. Moreso, the resistant nature of the thrombus also reduces the likelihood of a successful minimally invasive procedure such as AngioVac, which is typically reserved for fresh, soft, and malleable clots occurring within a couple of weeks (commonly 14 days). In addition, the patient's BMI (50 kg/m2), uncontrolled comorbidities (type 2 diabetes mellitus, COPD, sleep apnoea and obesity hypoventilation syndrome), and severe pulmonary hypertension (RVSP 94 mmHg) placed him at extremely high risk for an open surgical procedure, with the risk potentially outweighing the benefits. It was thus determined that continual anticoagulation therapy was the preferred method of management at the time, and he was transitioned to warfarin with the intention of lifelong therapy. He was also referred to the outpatient haematology clinic for possible workup of an underlying hypercoagulable state (in-patient workup is usually low yield and does not significantly impact management in the acute phase), the sleep clinic for sleep apnoea or OHS evaluation, the pulmonary clinic for evaluation of pulmonary hypertension, and a routine primary cardiology follow-up was established. It was planned that if his co-morbidities are optimised and it is determined that CTEPH is a significant contributor to his severely elevated pulmonary pressures, he may benefit from surgical management of the CTEPH at a specialised centre as well as further evaluation of his calcified right atrial thrombus with cardiac MRI and possible right heart catheterisation after sufficient anticoagulation if surgical extraction becomes an option.

## Conclusions

The detection of a calcified right atrial thrombus is unusual and may have diagnostic and therapeutic implications. Our systematic approach to this complex and unique case of a patient at high risk of surgery-related morbidity and mortality illustrates the invaluable role of a multidisciplinary approach to care. Furthermore, in the face of varying treatment options and the absence of clear guidelines, the importance of carefully reviewing and choosing a treatment strategy on a case-by-case basis cannot be overemphasised.
